# Supplementary material for: A new comprehensive eye-tracking test battery concurrently evaluating the Pupil Labs glasses and the EyeLink 1000
Source: PeerJ. 2019 Jul 9;7:e7086. doi: 10.7717/peerj.7086 (PMC6625505; doi:10.7717/peerj.7086)
Supplement: Supplemental Information 1 [file peerj-07-7086-s001.pdf]

SUPPLEMENTARY MATERIALS

Comment Sheet

Table 1. Summary of participant information. *ELC failure* indicates a validation error consistently greater than 0.5° for the EyeLink 1000 during the initial calibration. Likewise, *PLC failure* indicates a validation error consistently greater than 1.5° for the Pupil Labs glasses. Only participants printed in bold font were included in the analysis.

Subject	Sex	Age	Dominant Eye	Handedness	Eye Color	Exclusion reason
**1**	**f**	**27**	**right**	**right**	**blue green**	**-**
**2**	**f**	**24**	**left**	**right**		**-**
**3**	**f**	**21**	**right**	**right**	**blue**	**-**
**4**	**f**	**21**	**right**	**right**	**dark brown**	**-**
5	f	20	left			PLC failure
6	f	25	right			Experiment crash
7	m	28	right	right	blue	PLC failure
8	f	22	left	right	dark brown	Experiment crash
9	f	25	right			Early Interrupt
10	f	26	right			PLC and ELC failure
**11**	**m**	**24**	**left**	**right**	**light brown**	**-**
**12**	**m**	**21**	**right**	**right**	**light brown**	**-**
13	f	25	left		blue green	ELC failure
**14**	**f**	**24**	**right**	**right**	**blue**	**-**
**15**	**f**	**22**	**left**	**right**	**light brown**	**-**
16	f	25	left	right	blue	ELC failure
17	f	27	right	right	dark brown	ELC failure
18	f	22	right			Experiment crash
**19**	**m**	**29**	**right**	**right**	**dark brown**	**-**
**20**	**m**	**26**	**right**	**right**	**blue**	**-**
21	m	23	left	right	blue	Recording Problems
**22**	**m**	**28**	**right**	**right**	**blue**	**-**
**23**	**m**	**26**	**right**	**right**	**blue**	**-**
**24**	**f**	**25**	**right**	**right**	**blue**	**-**
**25**	**f**	**19**	**right**	**right**	**light brown**	**-**
**26**	**f**	**27**	**right**	**right**	**blue**	**-**
